# A Push–Pull Strategy to Enhance Biomass and Lipid Production in *Nannochloropsis oculata*

**DOI:** 10.3390/microorganisms13051131

**Published:** 2025-05-15

**Authors:** Roxana Guadalupe Tamayo-Castañeda, Gloria Viviana Cerrillo-Rojas, Teodoro Ibarra-Pérez, Christophe Ndjatchi, Hans Christian Correa-Aguado

**Affiliations:** Instituto Politécnico Nacional, Unidad Profesional Interdisciplinaria de Ingeniería Campus Zacatecas (UPIIZ), Zacatecas 98160, Mexicogcerrillor@ipn.mx (G.V.C.-R.); tibarrap@ipn.mx (T.I.-P.); mndjatchi@ipn.mx (C.N.)

**Keywords:** microalgae nitrogen-stress, lipid biosynthesis, phytohormone stimulation, auxins, cytokinin, Box–Behnken, optimization, biodiesel

## Abstract

The high demand for sustainable biodiesel feedstocks has led to the exploration of innovative strategies to enhance lipid productivity in microalgae. This study introduces a push–pull strategy to optimize lipid accumulation in *Nannochloropsis oculata*. The benzyl amino purine (BAP) and naphthalene acetic acid (NAA) stimulation, acting as the ‘push’ component, significantly boost growth and nutrient stress tolerance. Meanwhile, the ‘pull’ component, nitrogen (N) deficiency, triggers lipid biosynthesis. A Box–Behnken design was employed to optimize the factors named BAP fraction (0–1), total phytohormone (PH) BAP/NAA mix dose (0–20 ppm), and N-concentration (0–50%). The combined BAP/NAA treatment significantly increased biomass (15% higher than the control) and mitigated N-stress with higher doses (20 ppm). Lipid yield surged from 12.4% to 38.87% under optimized conditions (23.25% N, 39.5 ppm NAA, and BAP fraction 0). The push–pull strategy contributed to boosting lipid synthesis and balancing biomass production. N-limitation and total PH dosage were the determining factors in this strategy. This work demonstrates the potential of the push–pull strategy in increasing lipid accumulation, offering a promising and optimistic solution for biodiesel production at scale from microalgae. By reducing dependence on fossil fuels, *N. oculata* emerges as a reliable feedstock for oil extraction and biodiesel.

## 1. Introduction

Fossil fuels have been the basis of global social and economic development for a long time. Greenhouse gas (GHG) emissions are expected to peak in the next few years but must decline rapidly. The decisions made by consumers and government policies in the coming years will have profound implications for energy markets and the future of our planet, highlighting the potential gravity of the situation. By 2023, fossil fuels represented up to 80% of the energy mix, and demand for them has been increasing, as are concerns about the depletion of reserves of these finite resource [1]. There is a linear relationship between energy consumption, populational economic growth, and carbon dioxide emissions. Consequently, the current climate change problem is primarily due to the generation of greenhouse gases (GHGs) from burning fossil fuels. Therefore, a change in the energy model is essential, with a vision focused on creating greener fuels to achieve carbon neutrality while meeting the global demand for energy services. This new model must be based on the use of renewable energies. Biofuels are energetically sustainable, their stability and energy density are high, and they reduce emissions of atmospheric pollutants [2]. However, the voracious demand for energy and the high costs of raw materials for biofuel production make their use globally unfeasible, perpetuating the use of fossil fuels [3]. Biodiesel is a renewable alternative fuel capable of competing with petroleum diesel. Biodiesel is the only alternative fuel used directly in existing engines, as its density and cetane number are similar to those of conventional diesel. The production and burning of biodiesel reduces emissions of particulate matter, polycyclic aromatic hydrocarbons, carbon dioxide, and sulfur dioxide by up to 41% [4]. Biodiesel (BD) is a biofuel made from mono-alkyl esters of long-chain fatty acids, obtained mainly from vegetable oils. The most commonly used vegetable oils for biodiesel are palm oil (32%), soybean oil (26%), and canola oil (15%); the remaining 27% of this total corresponds to other raw materials, such as used vegetable oils, virgin vegetable oils, and animal fats [5]. BD production from vegetable oils or microalgal lipids requires a transesterification reaction. In this process, triglycerides react with alcohol (methanol or ethanol) in the presence of a catalyst (acid, base, or enzymatic) to form fatty acid methyl esters (FAMEs). This step makes BD production more expensive because of the energy demand for purification, catalyst recovery, and wastewater treatment. The challenges in BD production are the focus of researchers, addressing these high costs and environmental issues [6]. Producing biodiesel to substitute petroleum would negatively impact agri-food, leading to ethical dilemmas and enduring discussions about food and fuel.

Microalgae emerge as a beacon of hope in the biofuel dilemma. Among the various feedstocks considered for biofuel generation, microalgae stand out due to their high fatty acid content. The large-scale cultivation of microalgae not only produces high O_2_ ratios but reduces the atmospheric CO_2_ concentration without compromising the production of food, fodder, and other products derived from food crops [7,8]. The versatility of microalgae biomass, which can be converted into biodiesel, bio-oil, bioethanol, biohydrogen, and biomethane through thermochemical and biochemical processes, offers a promising and sustainable solution. This approach allows for the simultaneous and sustainable production of energy and specific high-value compounds under a biorefinery concept that integrates biomass conversion processes to produce fuels, power, and chemicals.

Microalgal biodiesel provides sufficient environmental advantages to merit subsidies. However, the main limitation is biomass recovery, which refers to collecting and extracting the microalgal biomass, and production costs compared to other feedstocks. Microalgal biomass recovery remains a major bottleneck due to high energy demands and costs. Current dewatering methods, such as centrifugation, are efficient but energy-intensive (~8 kWh/m^3^), limiting scalability [9]. Membrane filtration uses less energy but is prone to fouling and has high maintenance requirements [10]. The microalgal biomass flocculation is cost-effective, but it may contaminate the biomass. Emerging techniques like electro-coagulation and bio-flocculation show promise for sustainable harvesting [11].

The search for and selection of highly oil-producing strains and the development of optimized cultivation conditions to achieve competitive cost and maximum productivity in terms of microalgal biodiesel is urgent [12]. *Nannochloropsis* spp. has been studied as a biofuel feedstock for decades due to its high lipid productivity. The U.S. Department of Energy’s Aquatic Species Program (NREL, 1998) identified *Nannochloropsis* as a top candidate, and recent genomic and metabolic engineering advances have further validated its potential [13,14]. The potential of *Nannochloropsis* spp. as a biofuel feedstock is intriguing and should pique the audience’s interest. *Nannochloropsis oculata* has been shown to have high potential for biodiesel production: it has a high growth rate and CO_2_ fixation, a high lipid content, and a high yield. Additionally, it is easy to grow in saline, non-saline environments and under various pH and temperature conditions [15]. In addition, *N. oculata* possesses a high percentage of monounsaturated fatty acids, making it an ideal feedstock for biodiesel production [16].

While microalgae have long been considered promising feedstocks for biodiesel, the economics of microalgal biodiesel production need significant improvement. This challenge should serve as a call to action, motivating the audience to further conduct research and innovate in this field. Several strategies have been evaluated in order to boost microalgal biomass and lipids. For example, there are those based on the generation of abiotic stress, which refers to non-living factors in the environment that can increase the accumulation of lipid microalgae by modifying variables such as nitrogen (N), phosphorus (P), sulfur (S), pH, salinity, temperature, and light. Of all the above, N-deficiency is the most effective way of modifying the carbon storage pattern in a way that favors the synthesis of lipids, mainly triacylglycerol (TAG). In *Chlorella vulgaris*, a three-day N-deficiency caused an increase to 33.33% lipid content, with low biomass, photosynthetic pigment, and protein production [17]. In *Nannochloropsis oceanica*, gradual N-depletion for 7 days stimulated carbohydrate and TAG biosynthesis; the increase was 1.34- and 0.15-fold in carbohydrates and lipids, respectively. Although N-depletion stress strategies are a practical resource that favors lipid synthesis, they decrease cell division and growth, causing levels of low biomass production and, consequently, low lipid productivity [18,19]. This approach works well on a lab scale, but the application of more extensive systems, such as open ponds or photobioreactors, is challenging. In these larger systems, there are significant drawbacks, such as the uneven distribution of nutrients, higher chances of contamination, and lower overall biomass production (Baldev). Therefore, the need for more research and the development of innovative solutions to effectively address these challenges cannot be overstated [20].

On the other hand, studies on supplementing microalgal cultures with phytohormones (PH) have shown promising results in metabolic regulation. Singh et al. [21] evaluated the synergistic effect of auxins (indole acetic acid, indole butyric acid, and indole propionic acid) and cytokinins (benzyl amino purine and thidiazuron). They determined that treatment with the auxin indole butyric acid (AIB) increased cell size, while the use of cytokinin 6-benzyl amino purine (BAP) mainly promoted cell division in *Desmodesmus* sp. JS07, which favored biomass production and increased lipid content. BAP is a synthetic cytokinin containing four nitrogen atoms in its structure. It is presumed to act similarly in plants and microalgae, regulating cell division by activating histidine kinase (AHK) receptors and delaying senescence, though these mechanisms remain less understood. In microalgae such as *Nannochloropsis*, the absence of plant-like signaling components suggests that cytokinin BAP acts through partially conserved ancestral pathways, interactions with other hormonal systems (auxins, ethylene), or the direct modulation of metabolic enzymes [22]. However, other studies suggest that it enhances photosynthetic efficiency and carbon partitioning toward lipids [23]. In another study, the combined effect of indole acetic acid (IAA) and BAP on the growth of *Nannochloropsis* sp. were evaluated. The addition of auxins and cytokinins to the culture medium positively affected microalgae biomass production (3.65 g/L) and carbohydrate content (0.30 mg/L) [24]. Recently, this innovative approach to enhancing lipid production in microalgae piqued global scientific interest.

Other studies have revealed that applying PH under N-depletion improved lipid accumulation and maintained microalgal growth. This stimulant/stressing strategy, here defined as the push–pull type, could provide a way out of the bottleneck present in microalgal abiotic stress methodologies and strengthen those where only stimulation with growth regulators has been used. For example, the combined effect of 3-indole acetic acid (IAA) supplementation with N-depletion on *Nannochloropsis oceanica* was evaluated. The result was that combining IAA with N-depletion increased the biomass dry weight and the amount of fatty acids [25]. Another interesting study evaluated the effect of benzyl amino purine (BAP) and gibberellic acid (GA), coupled with N-limitation, on cell growth, biomass production, and fatty acid production in *Scenedesmus obliquus*. The results showed that BAP increased the biomass by 1.44-fold and GA by 1.35-fold. The total lipids increased by 2.8- and 1.11-fold. The phytohormone addition to *S. obliquus* at different initial nitrogen percentages of N0%, N25%, and N50% showed significant cell growth and biomass productivity compared to control cultures. N-starved BAP and GA induced 55% and 50% of the highest lipid yields [26].

The auxin naphthalene acetic acid (15 ppm) was applied to *Chlorella vulgaris* cultures under N- and P-deficiency conditions. This phytohormone did not show a significant effect on cell growth. However, it did stimulate the accumulation of neutral lipids in combination with N-limitation. Lipid content increased by 107% and doubled using 10 ppm NAA under simultaneous N- and P-limitation [27].

Nowadays, it is not only sufficient to use experimental strategies that improve the response of microalgae to one or more variable. If the objective is to determine the best operability conditions of a process, using optimization tools is indispensable to find the values that maximize lipid and biomass concentrations in microalgal cultures. The response surface methodology (RSM) overcomes the limitations associated with single-factor optimization. The Composite Central Design (CCD) and Box–Behnken design (BBD) are among the main types of response surface designs. The Box–Behnken design is a quadratic model, which can only be applied when three or more factors are used [28]. Few studies have used experimental designs with RSM to optimize microalgal cultures stimulated with phytohormones. One study used RSM to evaluate the effect of selenium (Se) and gibberellic acid (GA) on lipid content and biomass productivity in *Tetradesmus obliquus*.

Optimizing the dose of Se (15 ug/L) and GA (50 uM) increased the lipid content by 42.80%, with a biomass productivity of 0.964 g/L/d [29]. Using RSM, the combined effect of zeatin, IAA, and GA optimized biomass and microalgae lipid production in *Acutodesmus obliquus* under nitrogen limitation, where lipid content increased 1.9-fold over the control while maintaining biomass production [30]. The optimum conditions were 0.1 ppm zeatin, 1 ppm IAA, and five ppm GA. In another study, the combined effect of three phytohormones, myoinositol (MI), IAA, and abscisic acid (ABA), was assessed. Authors used RSM to analyze biomass production in *Dunaliella salina*. The 1.2-fold increase in biomass over the control was achieved with the optimized doses of 552 ppm MI, 0.14 M IAA, and 0.22 M ABA. The effects of phytohormone supplementation under nutrient depletion are species-dependent [31]. The individual behavior of microalgae species exposed to a particular phytohormone and the response to an applied stress strategy are arbitrary. The optimization of parameters between variables is crucial to increase the quantity and improve the quality of microalgal products. Consequently, cost reduction and better use of renewable resources would be achieved, contributing to biofuel production’s sustainability.

The objective of this research was to evaluate the effect of a mixture of the phytohormones 6-benzylaminopurine and naphthaleneacetic acid in combination with N-deficiency on lipid and biomass production of the microalga *Nannochloropsis oculata*. Using a Box–Behnken experimental design, the BAP fraction, the BAP/NAA mixture dose, and the N concentration were optimized to achieve higher lipid yields in biodiesel production while preserving microalgal growth.

## 2. Materials and Methods

*Nannochloropsis oculata* (UTEX 2164) was obtained from the algal collection of the University of Texas, Austin, TX, USA. Transparent 4 L glass vials were used with 3.5 L of Bold Basal Medium (BBM), with the optical density (750 nm) of *N. oculata* at 0.2. Cultures were incubated at 24 °C ± 2 °C, with light/dark cycles of 16/8 h. Fluorescent cool-white LED lamps (Tecnolite), set to an intensity of 65 µmol m^2^ s^−1^, were used. An aeration system was adapted with a flow rate of 2.5 L/min, with air continuously injected through a pump. The optical density of the culture was measured using a UV–Visible spectrophotometer (Thermo Scientific Genesys 10s, Waltham, MA, USA) at 750 nm (OD750) every 72 h to determine the growth kinetics. Once the early stationary stage was detected, the biomass was harvested by thecentrifugation of the medium at 4500 rpm for 10 min. The biomass was kept refrigerated (6 °C) until experimental assays were performed.

### 2.1. Experimental Design and Statistical Analysis

The experimental design for optimizing biomass and lipid production was carried out using a BBD in Design-Expert^®^ v11 Trial software for Windows (STAT-EASE^®^, Minneapolis, MN, USA).

In the experimental design, the independent variables were (1) the BAP fraction (A), (2) the BAP/NAA total dose (B), and (3) the N-concentration (C). Each factor has three concentration levels, which are shown in Table 1.

A block with five central points was adopted, with a design matrix of 17 runs (Table 2). The BAP fraction corresponds to the proportion of BAP present in the BAP/NAA growth regulator mixture. A BAP fraction equal to 1 indicates that only BAP was added to the culture medium (runs 1, 2, 5, and 7). A fraction of 0.5 represents an equimolar (1:1) mixture of BAP and NAA (runs 4, 6, 9–12, and 14–16). Finally, a fraction of 0 means only NAA was incorporated into the corresponding assay (runs 3, 8, 13, and 17). In addition, one run was kept as a control at a standard nitrogen concentration. Without phytohormone supplementation, this control was labeled as N100% (2.94 mM, 250 mg/L NaNO_3_ to the BBM standard). The response or dependent variable analyzed by the experimental design was lipid content (%).

The acquired data were fitted to a second-order polynomial model using Equation (1), which represents the model used to analyze the responses of the three-factor experimental design.

*y* = β0 + β_1_X_1_ + β_2_X_2_ + β_3_X_3_ + β_4_X_1_X_2_ + β_5_X_1_X_3_ + β_6_X_2_X_3_ + β_7_X_1_^2^ + β_8_X_2_^2^ + β_9_X_3_^2^ + ∑error (1)

where *y* is the response (% lipid); X_1_ = BAP fraction; X_2_ = Total BAP/NAA mixture dose; X_3_ = N-concentration (%); β0 = compensating term; β_1_, β_2_ and β_3_ = linear term coefficients; β_4_, β_5_, and β_6_ = coefficients for factor interaction; β_7_, β_8_ and β_9_ = quadratic effect coefficients; and ∑error = random error.

Once the experimental data were obtained, analysis of variance (ANOVA) was used, and the F-test was performed to establish the mathematical correlation between the inputs and outputs. *p*-values must be less than 0.05 to establish the significance of the model and its terms. Response surface and contour plots were used to visualize the dependence of the responses on each factor.

### 2.2. Nannochloropsis oculata Experimental Trials

The Bold Basal Medium [32] was prepared with different concentrations of N. The N-concentrations (%) of the BBM were N0% (0 mg/L, 0 mM), N25% (62.5 mg/L, 0.735 mM) N50% (125 mg/L, 1.47 mM), and N100%. The 250 mg/L (2.94 mM) sodium nitrate (NaNO_3_) in BBM was taken as N100%. The pH was adjusted to 8.0 with 1 N sodium hydroxide (NaOH) and autoclaved at 1 bar for 15 min. Working according to the experimental design (Table 2), 17 assays were performed by adding the medium with different N percentages and the corresponding phytohormone dosages. The assays were performed in sterile 1 L glass bottles. *N. oculata* was inoculated in 500 mL of BBM, and the D.O. of the medium was adjusted to 0.2 in a UV–visible spectrophotometer (Thermo Scientific Genesys 10s) at 750 nm.

Stock solutions of phytohormones were prepared at a concentration of 6000 ppm, sterilized in the autoclave, kept from light, and kept refrigerated until use (PhytoTechnology Laboratories^®^, Lenexa, KS, USA). Similar culture conditions were established to those described for the stock culture. Lipid and biomass concentrations were quantified periodically until an early stationary phase was detected.

### 2.3. Biomass Quantification

The dry weight (DW) biomass was estimated based on the linear relationship between OD750 UV–visible (Thermo Scientific Genesys 10s) and biomass g/L DW, which was obtained after multiple data analysis and calculated using Equation (2) [26]:

             Biomass (mg/L) = 729.36 (D.O.750) + 52.553 R^2^ = 0.9921 (2)

### 2.4. Lipid Quantification

The sulpho-phospho-vanillin (SPV) method was used for lipid quantification, as follows [33].

An aliquot (1 mL) was taken from the microalgae culture, and the cells were centrifugated at 5000 rpm for 10 min. The biomass was decanted and washed with deionized water (3 times with 2 mL) and dried at 100 °C until a constant weight was reached. The biomass (DW) was used for the SPV reaction. The lipid estimation was performed in a GENESYS 10S UV-Vis Spectrophotometer (Thermo Fisher Scientific, Waltham, MA, USA).

## 3. Results

**Figure 1:**
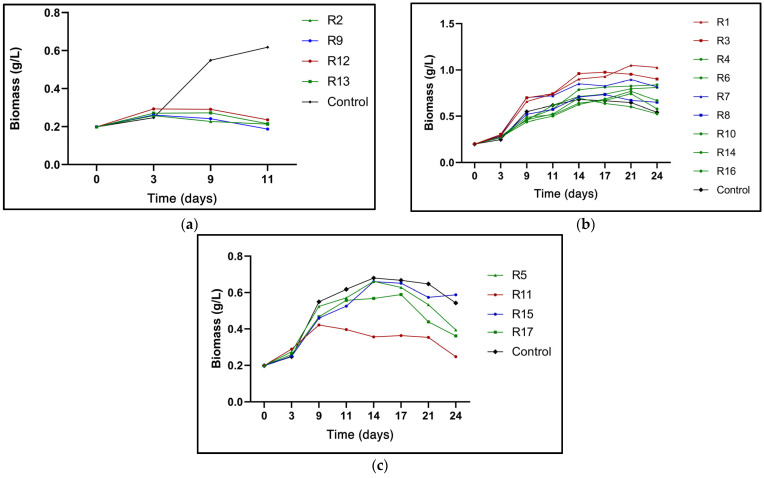
Biomass (g/L) in *N. oculata* calculated in early stationary phase of each treatment. (**a**): culture with N0% concentration; (**b**): culture with N25% concentration; (**c**): culture with N50% concentration. Blue lines: BAP/NAA total dose 5 ppm; green lines: BAP/NAA total dose 12.5 ppm; red lines: BAP/NAA total dose 20 ppm. A triangle (▲) in the connecting line of the graph represents the fraction of BAP = 1 and NAA = 0. The circle (●) shows the assays with the fraction of BAP = 0.5 and NAA = 0.5, and the square (■) is the fraction of BAP = 0 and NAA = 1 in the total dose of BAP/NAA mixture. The black color represents the control culture: N100% without PH stimulation.

The experimental design results were grouped according to nitrogen concentration (N0%, N25%, and N50%). Figure 1a–c shows the biomass production of all trials, including a control (N100%).

In Figure 1a, the lowest amounts of biomass were obtained under N0% concentrations in runs 2, 9, 12, and 13 (R2, R9, R12, and R13). The *N. oculata* cultures maintained a low but constant biomass production level for the first nine days. Adding the BAP/NAA mixture, a potential game-changer, significantly favored microalgae survival, offering a promising avenue for further research. However, on day 11, the ability of the phytohormones to alleviate nutrient stress in *N. oculata* declined as a considerable decrease in biomass was observed, accelerating the entry into the cell death phase. The potential of phytohormones to enhance microalgae survival in *N. oculata* is evidenced at a dose of 20 ppm (R12), characterized by higher production of dry cell weight (0.29 g/L) compared to lower doses of BAP/NAA (R2, R13: 12.5 ppm and R9: 5 ppm), which caused a slight decrease in the amount of biomass (Figure 1a). Data from the runs with phytohormone and N25% are shown in Figure 1b. The 20 ppm total doses of individual phytohormones addition, R1 (BAP = 1) and R3 (NAA = 1) consistently produced the highest amounts of biomass at the end of the evaluation (BAP: 1.049 g/L and NAA: 0.953 g/L). The mixture of both phytohormones in equal proportions at a total dose of 12.5 ppm also imparts a synergistic stimulatory effect on *N. oculata* cell division. The experimental central points R4, R6, R10, R14, and R16, all with an equimolar concentration of BAP/NAA at a total dose of 12.5 ppm, achieved a biomass of 0.685 ± 0.13 g/L. In trials R7 (BAP = 1) and R8 (NAA = 1), despite containing a low dose of PH (5 ppm), biomass production was higher than control biomass production, with values of 0.817 g/L, 0.651 g/L, and 0.542 g/L, respectively. The effect induced in the assays containing a BAP = 1 fraction was consistent; regardless of the total dose, BAP showed a more significant stimulus in biomass production than when using a NAA = 1 fraction (Figure 1b). In contrast to the N0% cultures (Figure 1a), those with a concentration of N25% could extend the early stationary phase until day 24 (Figure 1b). The results in Figure 1b show that a high BAP or NAA, R1, and R3 concentration is a determinant for extending the development of *N. oculata* cultures.

Regarding the cultures with N50% and phytohormones (Figure 1c), the total concentrations of 5, 12.5, and 20 ppm in the BAP/NAA mixtures R5, R11, R15, and R17 were not shown to increase biomass production compared to the control. Even the high dose of the BAP/NAA mixture at N50% appears to accelerate the aging of the *N. oculata* culture. For example, the R11 with 20 ppm BAP/NAA (1:1) N50% obtained approximately half the biomass (0.247 g/L) compared to the control (0.542 g/L). The R5 and R17 mixtures, with 12.5 ppm doses of FH (N50%), and R15 with its 5 ppm dose also showed lower final cell dry weight production than the control (Figure 1c). Despite R15 containing the lowest total PH concentration (5 ppm), it generated the highest biomass in N50% cultures, a behavior inverse to R11 (20 ppm). The results reveal that when N-deficiency is considerable (0–25%), the BAP/NAA mixture mitigates the cell nutrient stress. However, when the amount of N in the *N. oculata* culture is sufficient (50%), the PH-stimulating effect is detrimental at high doses and stimulating at low doses.

### 3.1. Effect of Push–Pull Strategy on Lipid Production

**Figure 2:**
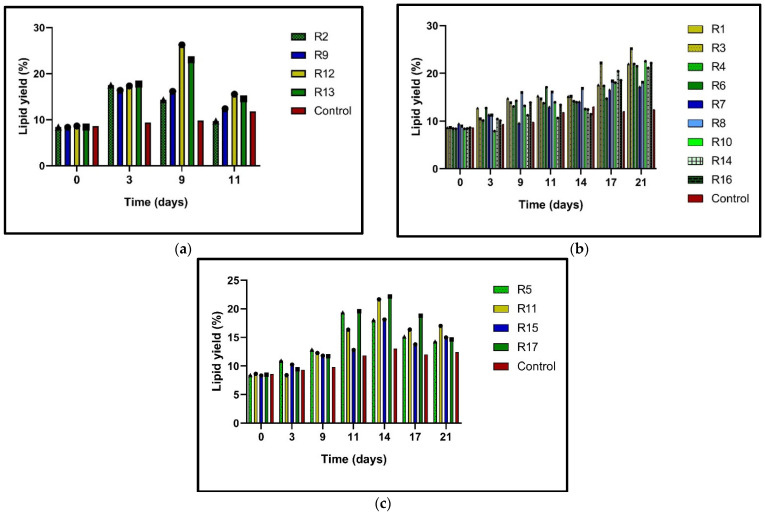
Lipid yield (%) in *N. oculata,* calculated in the early stationary phase of each treatment. (**a**): Culture with N0% concentration; (**b**): culture with N25% concentration; (**c**): culture with N50% concentration. Blue lines: PH total dose 5 ppm; green lines: PH total dose 12.5 ppm; yellow lines: PH total dose 20 ppm. The triangle (▲) in the connecting lines of the graph represents the fraction of BAP = 1 and NAA = 0. The circle (●) shows the assays with the fraction of BAP = 0.5 and NAA = 0.5, and the square (■) is the fraction of BAP = 0 and NAA = 1 in the total BAP/NAA mixture. The red color represents the control culture: N100% without PH stimulation.

Microalgae have been shown to increase their lipid content when subjected to stress conditions, including nitrogen limitation [34]. This increase in lipid content is significant as it can be harnessed for biofuel production and other industrial applications. The higher lipid content in microalgae under stress conditions means there is a higher potential for biofuel production, making this research particularly relevant for the biofuel industry. In this study, N-limitation (N0%, N25%, and N50%) in *N. oculata* cultures significantly stimulated lipid accumulation compared to the control (N100%) (Figure 2a–c). This increase in lipid content under stress conditions is promising for the biofuel industry, as it suggests that under certain conditions, microalgae can produce significantly higher lipid content, which can be converted into biofuel. The potential contribution of research to the biofuel industry is a significant implication of this study. In Figure 2a, the R2, R9, R12, and R13 N0% trials show significantly increased lipid content during the nine days of incubation; at the early stationary phase, a period of reduced growth was detected. The highest lipid percentages were obtained with the 20 ppm (R12) and 12.5 ppm (R13) doses. The R12 trial with a 20 ppm dose of BAP/NAA (1:1 equimolar) and the N0% treatment succeeded in accumulating 26.25% lipids in *N*. *oculata*, which a 2.11-fold higher value than exhibited by the control. Meanwhile, in R13 with the 12.5 ppm dose of NAA N0%, 25.19% was obtained, which was 2.03 times higher than in the control. In the case of the trials with N25% (Figure 2b), the highest result in lipid content was presented in R3 (20 ppm NAA). This stimulus obtained 25.19% lipid content, which was 2.03-fold greater than in the control. Similarly, the rest of the runs with 25% N and a 12.5 ppm phytohormone mixture of BAP/NAA (R4, R6, R10, R14, and R16) reached up to 22.5% lipid content. Finally, runs 5, 11, 15, and 17 obtained the lowest lipid ratios (Figure 2c). With N50% and PH, these cultures accumulated lipids at 14.28–16.98% rates on day 21. However, it is observed that day 14 was where the R11 and R17 N50% cultures presented the highest lipid percentages, ranging from 18.1 to 22.1%. All the N50% cultures with PH also presented a higher lipid accumulation level than the control. Figure 2c shows that on day 21, the R15 with a 5 ppm total dose (BAP+NAA) had the lowest content at 12.96%, while the highest yield was seen at the 20 ppm dose (BAP/NAA) with 16.98%. These data show that lipid biosynthesis is caused by N-deficiency and phytohormone stimulus, which is a significant finding for biofuel production.

### 3.2. Response Surface Methodology for Lipid N. oculata Optimization

A response surface design (RSM) was used in this study. Unlike factorial designs, where the best treatment is the “winner” of the total examined, RSM determines the optimal point, i.e., the best combination of the factors studied in the operability region. Using statistical and mathematical methods, RSM evaluates the effects of various control parameters on different system responses and identifies the best combination of these factors for optimizing system performance [28]. The Box–Behnken design (BBD) is a rotating or quasi-rotating second-order response surface methodology. It is based on three-level incomplete factorial designs and was specifically designed to establish cause-and-effect relationships between factors and responses in experiments. Unlike the CCD, the BBD does not simultaneously contain combinations with factors at higher or lower levels, ensuring that measurements are not taken in extreme situations [35]. The RSM is a powerful tool that can predict the PH and N effects on lipid production in *N. oculata* and optimize these responses, offering a promising future for research in this field.

Table 3 shows the Box–Behnken design variables and results obtained for biomass and lipid content in *Nannochloropsis oculata*. The independent variables were BAP fraction (ppm) (A), total doses of BAP/NAA (ppm) (B), and N-concentration in culture (C). Regression equation coefficients were calculated, and the data were fitted to a second-order polynomial equation for lipid and biomass production.

The second-order quadratic model obtained is shown below (Equation (3)).

X = 21.84 − 1.65 × A + 3.00 × B − 2.36 × C − 0.5550 × AB + 2.09 × AC − 2.01 × BC − 1.62 × A^2^ + 0.4215 × B^2^ − 3.67 × C^2^ (3)

The coded factors are X = lipid yield, A = fraction of BAP in the mixture, total dose (BAP/NAA), and C = nitrogen concentration.

According to the analysis of variance (ANOVA) for lipid yield, the model is statistically significant as it presents a significance level *p*-value of 0.0406 (<0.05) and an F-value of 4.00 (>1). A significance level (*p*-value) of 0.05 indicates that the probability of the model not fitting the data is only 5%. Also, the lack of fit is insignificant, with a *p*-value of 0.795 (>0.05) and an F-value of 0.3461 (<1). A non-significant lack of fit indicates that the model adequately describes the functional relationship between the experimental factors and the response variable. The model fit is good and can be used as a predictor of responses (Table 4). *p*-values < 0.05 indicate that the model terms are significant; in this case, only factors A, B, C, A^2^, and C^2^ are significant. The linear terms AC and BC are significant for the model. The ANOVA and response surface design results revealed how BAP/NAA and N influence lipid accumulation in *Nannochloropsis oculata*. For example, factor A (fraction of BAP in the mixture) had significance for lipid accumulation (*p*-value 0.0008). The value of the quadratic term A^2^ (*p* = 0.0049) indicates the existence of a non-linear relationship, i.e., very high or very low proportions of BAP reduce lipid production. The A^2^ value suggests that there is a balance between cell division and metabolic stress. Table 4 shows that factor B had the strongest effect (*p* < 0.0001, SS = 71.82). This result indicates that the BAP/NAA mixture is effective in treatments under N-stress conditions, mitigating the accelerated cell death of *N. oculata*. BAP likely promoted cellular division while maintaining biomass production under N-limitation, while NAA induced higher tolerance to nutrient stress. Consistent with our study, various combinations of auxins and cytokinins mitigated oxidative stress in microalgae and increased lipid production [25,36]. Factor C also indicated that there was a significant effect (*p* < 0.0001, SS = 44.46). At the same time, the quadratic term C^2^ (*p* < 0.0001) indicates the existence of a non-linear optimal response. With the above, the model explains that N-limitation induces stress and stimulates the response towards lipid production. However, under N0% conditions, the most substantial effect is directed towards cell death. A previous study reported that *N. oceanica* supplementation with moderate N percentages mainly increased TAG synthesis by 51% while maintaining biomass production [37].

### 3.3. Effect of Model Terms on Biomass Production

**Figure 3:**
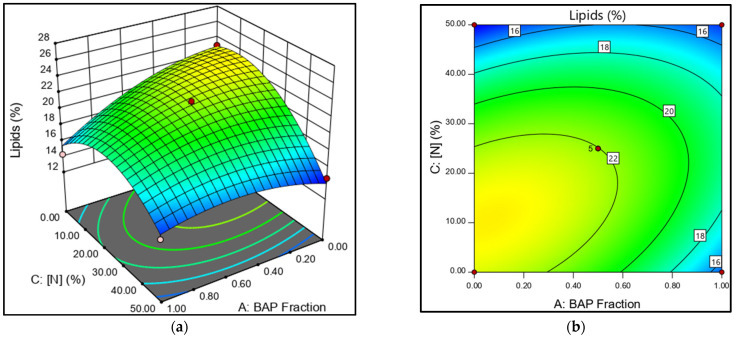
(**a**) Three-dimensional response surface plot. (**b**) Contour plot. A: BAP fraction and C: N-concentration effects on total lipid yield in *N. oculata*.

The 3D response surface plot, a graphical representation of the relationship between two factors and a response, shows the effect of phytohormone concentration on lipid production. According to the ANOVA analysis, the mixture dose (BAP/NAA) and the N-concentration are the most significant factors. A response surface plot is presented to analyze the role of the AC interaction (Figure 3a,b). The AC factor shows that the BAP effect depends on the level of N. For example, under high N-concentrations (N50%), a higher proportion of BAP improved biomass production; however, this generated a lipid reduction. At the N25% concentration and low BAP ratios, lipid production increased in *N. oculata*. The contour plot (Figure 3b) shows lipid production in relation to N and PH concentrations, further illustrating this relationship.

**Figure 4:**
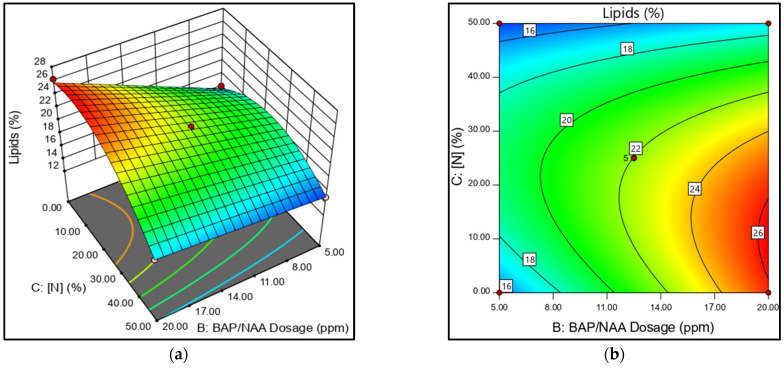
(**a**) Three-dimensional response surface plot. (**b**) Contour plot. B: BAP/NAA dosage and C: nitrogen concentration effects on yield of total lipid in *N. oculata*.

The best lipid yields were obtained with N-limitation (Figure 4a,b). The *N. oculata* medium, with N25%, presented results that were consistent with high lipid production. Meanwhile, cultures with N0% presented high and low values depending on the total dose of PH applied. However, the early cell death in the N0% cultures causes the high cell lipid production to be unbalanced with low biomass, even with the PH stimulus. Figure 4a shows the inverse relationship in N50% cultures. As N values are raised, the lipid yield decreases. In this case, no significant effect of the applied phytohormone dose was observed on lipid yield (Figure 4b).

The combined effect of BAP/NAA was expected to have a higher stimulatory effect on biomass and lipid production than their individual effects. The trials with 12.5 ppm of the NAA+BAP mixture showed stimulatory effects on biomass and lipid production (Figure 3 and Figure 4). However, looking at the individual effect of BAP and NAA at a concentration of 20 ppm with N25%, a high lipid yield was obtained with NAA (25.19%)and the highest biomass production was obtained with BAP (1.049 g/L).

## 4. Discussion

This study demonstrated the potential impact of exogenous phytohormone addition to microalgal culture, which could inspire further researchin the field. The biochemical effects of a mix of phytohormones, such as BAP and NAA, on specific microalgae species have not been fully explored. Furthermore, the functional role of BAP and NAA in the oleaginous *N. oculata* lipid production under N-limited conditions presents a promising area for future investigation.

The absence of N causes lower biomass productivity because the limitation of this nutrient decreases cell growth to conserve energy and nutrients [38]. Nitrogen is a significant component in the construction of chloroplasts and chlorophyll synthesis, and so nitrogen depletion leads to chloroplast decomposition [39]. BAP and NAA addition to *N. oculata* (N25%) as single doses of 20 ppm showed the highest biomass production on day 21 of incubation, with values of 1.049 g/L and 0.953 g/L, respectively (Figure 1b). It was found that cytokinins are key in the cell signaling process of N in microalgae. The addition of the cytokinin BAP to *Desmodesmus* sp. cultures exerted a more significant stimulus (push) on biomass production (1.8 g/L) than the auxins indole-3-acetic acid (IAA) (1.3 mg/L) and indole-3-propionic acid (IPA) (1.44 g/L) at a concentration of 10 ppm. The stimulatory effect was attributed to the fact that cytokinins in microalgae stimulate key enzymes involved in nitrogen assimilation and amino acid synthesis, consequently enhancing cell growth and division [21]. In another study, the addition of BAP to *Chlamydomonas* was associated with an increase in cell number by enhancing the photorespiration process and elevating mRNA levels and serine-glyoxylate aminotransferase (SGAT) activity [40]. It is reported that cytokinins influence cellular carbon and nitrogen metabolism by stimulating the activity of the nicotinamide adenine dinucleotide (NADH)-dependent enzyme hydroxy pyruvate, increasing the production of photosynthetic pigments. Likewise, cytokinins such as BAP can stimulate NADH-dependent glutamate dehydrogenase activity [41]. In *Chlorella pyrenoidosa*, the effect of 4 phytohormones—BAP, 6-furfuryl amino purine (Kinetin), GA, NAA, and indole-3-butyric acid (IBA)—on the yields of biomass and α-linolenic acid were studied. The result was that only NAA and BAP induced high biomass yields of 2.2-fold and 1.26-fold, respectively [42]. Contrary to our results, NAA responded better than BAP in *C. pyrenoidosa*. It is well known that the response or effect of a growth regulator is species-specific.

On the other hand, auxins such as NAA have shown a considerable effect on the growth rate of microalgae in several studies [43,44]. NAA and other phytohormones were added to *B. braunii* B12 to evaluate their effect on biomass and carotenoid production. The most effective phytohormone was NAA (50 mg/L) in stimulating cell growth and carotenoid accumulation. Cell growth increased two-fold compared with that of the control [45].

The combined effect of BAP and NAA had two behaviors. At higher doses of the mixture (20 ppm) and N25%, the effect was stimulatory on biomass, while at the same dose of the mixture and N50%, the effect was inhibitory (Figure 1c). BAP and NAA (N0% and N25%) can activate mechanisms that mitigate nutrient stress to achieve crop growth at low N-concentrations. Cytokinins and auxins have been shown to alleviate oxidative stress in N-limited microalgal cells by positively regulating the activity of antioxidant enzymes. Increased antioxidant activity decreases the generation of reactive oxygen species (ROS) and cell damage. BAP is a potent phytohormone that promotes ROS scavenging. For example, BAP strongly activated ROS scavenging enzymes catalase (CAT) and ascorbate peroxidase (APX) [21,46]. Similarly, auxin AIA was shown to increase peroxide dismutase (SOD) activity to a greater extent than CAT and APX in *Acutudesmus obliquus* [46]. Earlier studies revealed that BAP and NAA can used as growth regulators to promote microalgal cell growth and enlargement and boost lipid accumulation [24,47].

However, the inhibitory effect observed in N50% cultures spiked with the BAP/NAA mixture at 20 ppm is likely because high doses of cytokinins block cell proliferation and induce programmed cell death (PCD). In *Arabidopsis* plants, high doses of cytokinins have been observed to induce programmed cell death (PCD) in proliferating cells [48]. One of the most intriguing aspects of our study is the dose-dependent nature of the paradoxical effect of auxins. Depending on the dose, auxins can either stimulate or inhibit the development of *N. oculata.* A high dose of phytohormones, combined with a sufficient N-concentration (50%), was the key factor triggering the inhibitory effect. The dose-dependent paradoxical effect of auxins is a fascinating area for further research.

N-limitation reduces biomass productivity to conserve energy and nutrients [49]. This study’s experimental data showed that N-limitation caused a higher level of lipid production compared to the control. Nitrogen deficiency induces most of the carbon fixed by photosynthesis to modify its pathway towards lipid synthesis. This mechanism increases lipid content and decreases carbohydrate content, which reduces cell division [50]. The decrease in cell division alters the lipid biosynthetic pathway, allowing us to accumulate more saturated and unsaturated lipids to resist cellular stress [51].

ANOVA analysis shows that factor B (BAP/NAA dose mixture) significantly affected lipid production. As B increases, lipid concentration increases. As exogenous, additives, FHs are important for lipid accumulation. Plant hormones promote cell growth and lipid accumulation under stress conditions [52]. Similar to this study, NAA has been reported to affect lipid synthesis positively. NAA participated in lipid biosynthesis regulatory signals of *Chlorella vulgaris* by modifying the content of endogenous indole-3-acetic acid, jasmonic acid, and salicylic acid. NAA (1 ppm) generated 47% lipid content in *C. vulgaris* microalgae [47]. In *Scenedesmus* sp. and *Chlorella sorokiniana*, a mixture of two auxins at a total dose of 20 mg/L (10 mg/L NAA + 10 mg/L IBA) increased lipid content 2.4 and 2.9-fold over the control (no phytohormones) under nitrogen-limiting conditions [39].

In this work, factor C (N-content) was one of the primary growth parameters influencing lipid production (Table 4). *N. oculata* N0% and phytohormones doubled lipid production compared to the control (Table 3). A dose of PH under nitrogen depletion may reinforce the prioritization of carbon flow for lipid synthesis. PH increases the expression of genes such as *accD* (acetyl-CoA carboxylase heteromeric beta subunit (ACCase)), which is involved in carbon fixation and lipid biosynthesis, with acetyl-CoA carboxylase being a key enzyme in lipid biosynthesisb [53]. When evaluating the effect of N-concentration on lipid content in *N. oculata* without phytohormone stimulation, a concentration of N25% (75 mg/L) doubled the lipid content from 7.9 to 15.8% and increased lipid content 1.9-fold compared to the control. A decrease in lipid content was observed when factor C was increased to N50% and factor B was increased to the maximum dose (20 ppm) (Figure 2a). Similarly, in *Desmodesmus* sp. JS07, the addition of a total dose of 15 ppm of a mixture of BAP and IBA (indole 3-butyric acid) caused high lipid production. However, a dose of 20 ppm decreased the lipid yield [21]. In congruence with our results, a 12.5 ppm dose generated higher percentages of lipids than 20 ppm doses (Figure 2b). Increasing the dose of phytohormones can decrease lipid production in microalgae. For example, in *Graesiella emersonii* and *Chlorophyta* sp., the effect of N-limitation was analyzed in cultures supplemented with a mixture of indole acetic acid (IAA) and kinetin (K) at 10 ppm, 20 ppm, and 30 ppm. The results showed that a 20 ppm dose generated the highest lipid productivity, while a 30 ppm dose decreased the lipid yield [54].

Based on the above, the push–pull strategy (phytohormone stimulation/N-limitation) is suitable for stimulating lipid biosynthesis in *N. oculata*. This methodology increased biomass production, decreased N-concentrations in the medium, and significantly increased lipid content in biodiesel production. We selected a solution to maximize lipid production. This allowed us to optimize the target response (% lipids). The proportion of BAP was minimized, the total PH dose concentration was maximized, and a range of N0% to N25% was maintained. The total PH dose limit was 40 ppm (Table 5).

The results of the optimization and experimental validation are shown in Table 6. The application of auxins such as indole acetic acid (IAA), indole butyric acid (IBA), and indole propionic acid (IPA) in *Chlorella pyrenoidosa* and *Scenedesmus quadricauda* from 5 to 60 ppm showed that, from 40 to 60 ppm lipid production, was higher than that at lower concentrations [41]. According to the optimization table, with a concentration of 39.5 ppm NAA and 23.25% N, the % lipid increased to 38.87 ± 0.927%.

The experimental value (38.87 ± 0.927%) showed a statistically significant difference from the model prediction (40.915%) (*t*-test, t = 4.41, *p* = 0.021). However, the model maintained its practical validity because the relative error (4.99%) was within acceptable limits for biological processes. Error results of less than 10% are considered statistically acceptable [55]. Furthermore, the predicted R^2^ = 0.8566 exceeds the reliability threshold (>0.8), affirming the model’s suitability for biodiesel production optimization.

It is known that discrepancies between predicted and experimental values in microalgal evaluations are common due to metabolic complexity, cellular heterogeneity, nonlinear responses to environmental stimuli, and variability in nutrient uptake [56,57]. These variations are unavoidable and do not invalidate the usefulness of the present model.

## 5. Conclusions

The push–pull strategy’s efficacy in optimizing lipid production in *Nannochloropsis oculata* was demonstrated, resulting in a substantial increase from 12.4% to 38.87%. This significant improvement underscores the success of the strategy. The effect of phytohormone addition (BAP and NAA), even under conditions of nitrogen limitation, improved biomass productivity, highlighting the potential of this approach to balance growth and lipid accumulation. Nitrogen deficiency and phytohormone dosing play crucial roles in redirecting metabolic pathways toward lipid biosynthesis, and so many avenues remain open for metabolic and other stressor research applying the push–pull strategy. This strategy holds great promise for industrial applications, especially in developing sustainable biorefineries. The environmental advantages of using microalgae as a renewable feedstock make this work a cornerstone for advancing green technologies.

## Figures and Tables

**Table 1:** Factors and levels for the experimental design for the optimization of phytohormone (BAP/NAA) and nitrogen concentration in *N. oculata* cultures.

Factors	Factor Code	Levels	
		Low	High
BAP fraction	A	0	1
BAP/NAA total dose (ppm)	B	5	20
N concentration (%)	C	0	50

BAP: benzyl amino purine. NAA: naphthalene acetic acid. N: nitrogen.

**Table 2:** Box–Behnken design matrix with 17 runs using a quadratic model.

Run	BAP Fraction	BAP/NAA Total Dose (ppm)	N (%)
1	1	20	25
2	1	12.5	0
3	0	20	25
4	0.5	12.5	25
5	1	12.5	50
6	0.5	12.5	25
7	1	5	25
8	0	5	25
9	0.5	5	0
10	0.5	12.5	25
11	0.5	20	50
12	0.5	20	0
13	0	12.5	0
14	0.5	12.5	25
15	0.5	5	50
16	0.5	12.5	25
17	0	12.5	50
Control	0	100	0

BAP: benzyl amino purine. NAA: naphthalene acetic acid. N: nitrogen.

**Table 3:** Box–Behnken design matrix with 17 runs using a quadratic model.

Run	BAP Fraction	BAP/NAA Dose (ppm)	N (%)	Lipids (%) Observed	Lipids (%) Predicted
1	1	20	25	22.01	21.43
2	1	12.5	0	14.31	15.17
3	0	20	25	25.19	25.84
4	0.5	12.5	25	21.95	21.84
5	1	12.5	50	14.28	14.63
6	0.5	12.5	25	21.49	21.84
7	1	5	25	17.20	16.55
8	0	5	25	18.16	18.74
9	0.5	5	0	16.17	15.95
10	0.5	12.5	25	22.51	21.84
11	0.5	20	50	17.02	17.22
12	0.5	20	0	26.26	25.96
13	0	12.5	0	23.03	22.65
14	0.5	12.5	25	21.12	21.84
15	0.5	5	50	14.96	15.26
16	0.5	12.5	25	22.14	21.84
17	0	12.5	50	14.63	13.76
Control	0	100	0	12.4	

BAP: benzyl amino purine. NAA: naphthalene acetic acid. N: nitrogen.

**Table 4:** Analysis of variance for response surface quadratic model to study BAP fraction, BAP/NAA dosage, and N-concentration on lipid yield in *N*. *oculata*.

Source	Sum of Squares	df	Mean Square	F-Value	*p*-Value
Model	243.48	9	27.05	39.98	<0.0001
A	21.75	1	21.75	32.14	0.0008
B	71.82	1	71.82	106.14	<0.0001
C	44.46	1	44.46	65.71	<0.0001
AC	17.43	1	17.43	25.76	0.0014
BC	16.20	1	16.20	23.94	0.0018
A^2^	11.10	1	11.10	16.40	0.0049
C^2^	56.59	1	56.59	83.63	<0.0001
Residual	4.74	7	0.6766		
Lack of Fit	3.56	3	1.19	4.06	0.1048
Pure Error	1.17	4	0.2929		

A: BAP fraction. B: BAP/NAA total dosage. C: nitrogen concentration.

**Table 5:** Levels and factors for the optimization of lipid yield in *N. oculata*.

Name	Goal	Lower Limit	Upper Limit	Lower Weight	Upper Weight	Importance
A	minimize	0	1	1	1	3
B	is in range	20	40	1	1	3
C	is in range	0	25	1	1	3
Lipids	maximize	14.28	26.26	1	1	5

A: BAP fraction. B: BAP/NAA dosage. C: nitrogen concentration.

**Table 6:** Theoretical model validation results against experimental values in the push–pull strategy for *N. oculata* lipid yield optimization.

BAP Fraction	BAP/NAA Dosage	% N	Theoretical Lipid Yield *	Experimental Lipid Yield **	% Error
**0**	39.500	23.249	40.915 ± 0.82	38.87 ± 0.927	4.99

* Mean ± CI (confidence interval, 95%); ** Mean ± SD (standard deviation, *n* = 3).

## Data Availability

The original contributions presented in this study are included in the article/Supplementary Materials. Further inquiries can be directed to the corresponding author.

## Supplementary Materials

The following supporting information can be downloaded at: https://www.mdpi.com/article/10.3390/microorganisms13051131/s1, Table S1: The components of BBM medium; Table S2: The composition of A5 solution for BG11; Table S3: Datos de curva de calibración de biomasa (n = 3) [58]; Table S4: Datos de curva de calibración de lípidos (n = 3); Figure S1: The method to determine the biomass curve calibration; Figure S2: Calibration curve of biomass; Figure S3: Calibration curve of lipids (SPV method); Figure S4: Color developed by SPV method in calibration tubes. (the picture was taken after the measurements, not all concentrations evaluated are shown).

## Abbreviations

The following abbreviations are used in this manuscript:

ABA	abscisic acid
BAP	benzyl amino purine
BBD	Box–Behnken design
BBM	Bold Basal Medium
CCD	Central Compose Design
GA	gibberellic acid
IAA	indole-3-acetic acid
IBA	indole-3-butyric acid
NAA	Naphthyl Acetic Acid
MI	Myositol
RSM	response surface methodology
ROS	reactive oxygen species
