# Supplementary material for: A Push–Pull Strategy to Enhance Biomass and Lipid Production in Nannochloropsis oculata
Source: Microorganisms. 2025 May 15;13(5):1131. doi: 10.3390/microorganisms13051131 (PMC12114038; doi:10.3390/microorganisms13051131)
Supplement: Supplementary file 1 [file microorganisms-13-01131-s001.zip › microorganisms-3545553-supplementary.pdf]

Supporting Information

A push-pull strategy to enhance biomass and lipid production in *Nannochloropsis oculata*

*Roxana Guadalupe Tamayo-Castañeda, Gloria Viviana Cerrillo-Rojas, Teodoro Ibarra-Pérez, Christophe Ndjatchi and Hans Christian Correa-Aguado[1] **

Instituto Politécnico Nacional, Unidad Profesional Interdisciplinaria de Ingeniería Campus Zacatecas (UPIIZ), Zacatecas 98160, México.

*Correspondence: hcorreaa@ipn.com

Corresponding Author:

* Hans Christian Correa-Aguado Ph. D

S1. *Nannochloropsis oculata* culture

Table S1. The components of BBM medium.

Medium Components	Stock solution （g/L dH_2O）	Dosage （mL /L）
$NaNO_3$	25.00	10
$CaCl_2 \cdot 2H_2O$	2.50	10
$MgSO_4 \cdot 7H_2O$	7.50	10
K_2HPO_4	7.50	10
KH_2PO_4	17.50	10
NaCl	2.50	10
Alkaline Solution		1
$EDTANa_2$	50.00	
KOH	31.00	
Acidified solution		1
$FeSO_4 \cdot 7H_2O$	4.98	
H_2SO_4		1
Boron solution		1
H_3BO_3	11.42	
Trace Metals solution		1
$ZnSO_4 \cdot 7H_2O$	8.82	
$MnCl_2 \cdot 7H_2O$	1.44	
MoO_3	0.71	
Cu $SO_4 \cdot 5H_2O$	1.57	
$Co(NO_3)_2 \cdot 62H_2O$	0.49	

S2. Determination of the calibration curve biomass vs OD_{750}

Four 1 L samples (in triplicate) of *N. oculata* were prepared and adjusted to different ODs (0.1, 0.2, 0.3, and 0.4). OD determination was performed: a known amount of microalgal suspension was sonicated (Branson M1800 CPX-952-116R) 2 cycles of 1 min, 30 s, 15 kHz, 30% amplitude. The suspension was then vortexed (3,000 rpm) for an additional 30 s. The suspension was read in a UV-Visible spectrophotometer

(Thermo Scientific Genesys 10s) at 750 nm. A filter paper (Whatman, 2.5 µm) was brought to constant weight in an oven at 105 °C for 24 h, and the filter dry weight (P0) was measured. The samples were filtered and dried in the oven at 50 °C for 24 h, and the filter paper with the biomass (P1) was weighed again. The biomass dry weight (BW) was obtained using equation 1.

$$PB\ (mg/L) = P1 - P0 \qquad eq.1$$

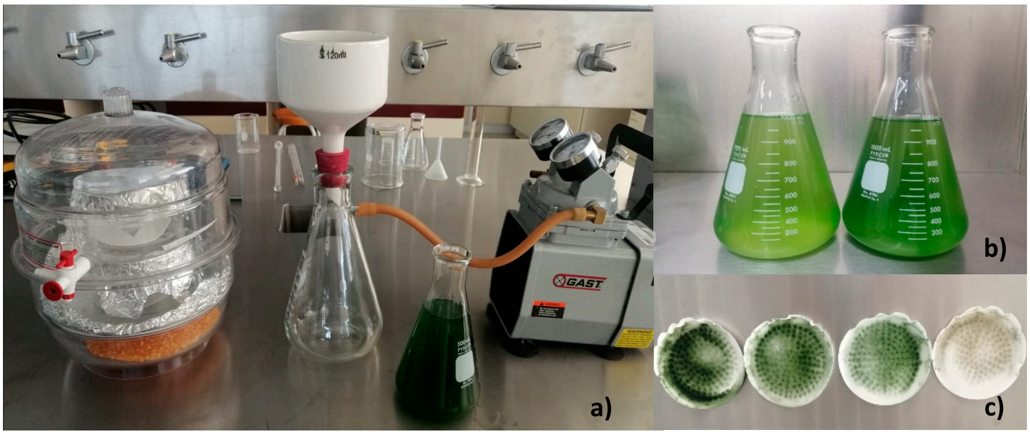

Figure S1. The method to determine the biomass curve calibration (n = 3).

Table S2. Biomass curve calibration and OD data

Trial	OD 750 nm	Biomass (mg/L)
1	0.100 ± 0.001	120.20 ±1.0
2	0.200 ± 0.010	215.71 ± 5.0
3	0.300 ± 0.005	263.20 ± 2.5
4	0.400 ± 0.008	351.40 ± 4.5

OD: optical density. (n =3)

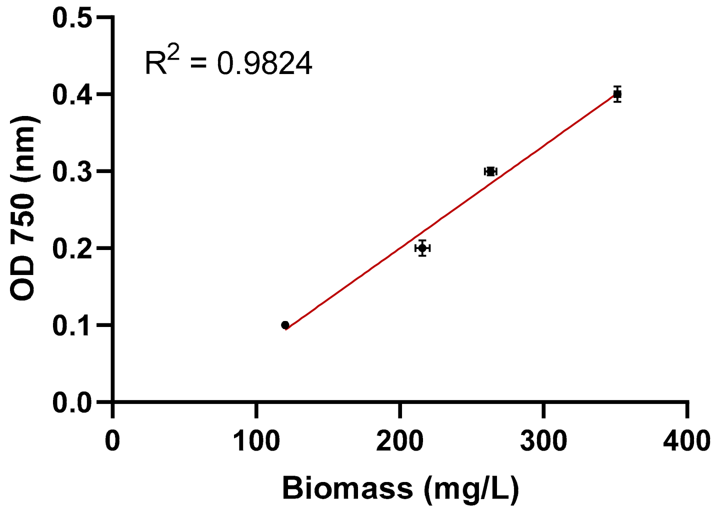

Figure S2. Calibration curve of OD vs biomass

S3. Determination of the lipid's calibration curve (SPV method)

Lipid quantification in *N. oculata* cultures was performed by a colorimetric method, using sulphophosphovanillin (SFV) according to the protocol of Mishra et al., 2014.

Table S3. Lipid calibration curve data (n = 3)

Test tube	Canola Oil (µg)	Absorbance 530 nm
1	5	0.004 ± 0.001
2	10	0.016 ± 0.005
3	15	0.041 ± 0.003
4	20	0.062 ± 0.002
5	30	0.098 ± 0.003
6	40	0.153 ± 0.003
7	50	0.204 ± 0.001
8	60	0.263 ± 0.005
9	70	0.325 ± 0.001

10	80	0.39 ± 0.005
11	100	0.434 ± 0.007
12	120	0.562 ± 0.008
13	160	0.742 ± 0.003
14	200	0.992 ± 0.007

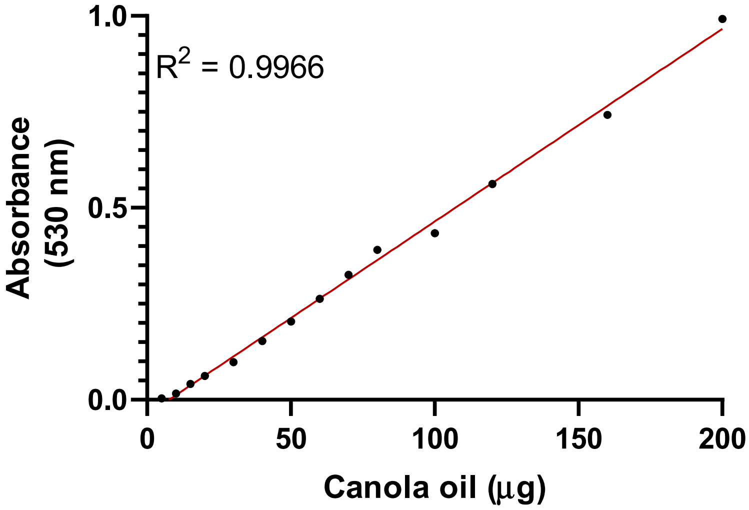

Figure S3. Calibration curve of lipids (SPV method)

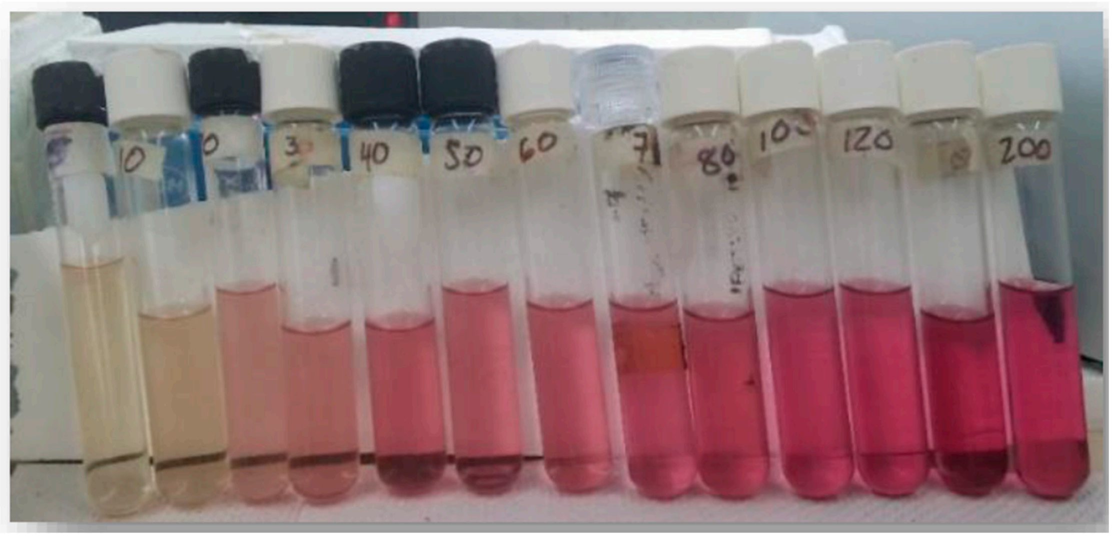

Figure S4. Color developed by SPV method in calibration tubes (the picture was taken after the measurements, not all concentrations evaluated are shown in figure).

S4. T-test validation

Table S4. Data to perform the T-test validation (n=3)

Theoretical Lipid Yield*	Experimental Lipid Yield**	% Error
40.915 ± 0.82	38.87 ± 0.927	4.99

Hypotheses:

(H_0): No significant difference between the theoretical and experimental means.

(H_1): There is a significant difference between the theoretical and experimental means.

T-test formula for two independent samples

$$t = \frac{(\bar{X}1 - \bar{X}2)}{\sqrt{\frac{s_1^2}{n_1} + \frac{s_2^2}{n_2}}}$$

$\bar{X}1$ and $\bar{X}2$ are the means of the two samples (theoretical and experimental).

s_1 and s_2 are the standard deviations of the two samples.

n_1 and n_2 are the sample sizes (n=3).

$t = \frac{(40.915 - 38.87)}{\sqrt{\frac{0.82^2}{3} + \frac{0.927^2}{3}}} = 2.86$

For the T-test with equal sample sizes (n=3), the degrees of freedom was calculated as:

$df = n_1 + n_2 - 2 = 4$

Critical value of the T-test**.**

Using a T-distribution table for df=4 and a significance level of α = 0.05, the critical value for a two-tailed test is approximately $t_{0.025,4}=2.776$.

The calculated t value is 2.86, greater than the critical value 2.776. Therefore, we reject the null hypothesis, indicating that there is a statistically significant difference between the theoretical and experimental lipid yields. However, the model maintained its practical validity because the relative error (4.99%) is within acceptable limits for biological processes. Less than 10 % error results are considered statistically acceptable.
